# Influence of high glucose and advanced glycation end-products (ages) levels in human osteoblast-like cells gene expression

**DOI:** 10.1186/s12891-016-1228-z

**Published:** 2016-08-31

**Authors:** Cristina Miranda, Mercè Giner, M. José Montoya, M. Angeles Vázquez, M. José Miranda, Ramón Pérez-Cano

**Affiliations:** 1 Bone Metabolism Unit, Internal Medicine Department, Virgen Macarena University Hospital, Dr. Fedriani s/n, 41009 Seville, Spain; 2 Medicine Department, University of Seville, Dr. Fedriani s/n, 41009 Seville, Spain

## Abstract

**Background:**

Type 2 diabetes mellitus (T2DM) is associated with an increased risk of osteoporotic fracture. Several factors have been identified as being potentially responsible for this risk, such as alterations in bone remodelling that may have been induced by changes in circulating glucose or/and by the presence of non-oxidative end products of glycosylation (AGEs). The aim of this study is to assess whether such variations generate a change in the gene expression related to the differentiation and osteoblast activity (OPG, RANKL, RUNX2, OSTERIX, and AGE receptor) in primary cultures of human osteoblast-like cells (hOB).

**Methods:**

We recruited 32 patients; 10 patients had osteoporotic hip fractures (OP group), 12 patients had osteoporotic hip fractures with T2DM (T2DM group), and 10 patients had hip osteoarthritis (OA group) with no osteoporotic fractures and no T2DM. The gene expression was analyzed in hOB cultures treated with physiological glucose concentration (4.5 mM) as control, high glucose (25 mM), and high glucose plus AGEs (2 μg/ml) for 24 h.

**Results:**

The hOB cultures from patients with hip fractures presented slower proliferation. Additionally, the hOB cultures from the T2DM group were the most negatively affected with respect to RUNX2 and OSX gene expression when treated solely with high glucose or with high glucose plus AGEs. Moreover, high levels of glucose induced a major decrease in the RANKL/OPG ratio when comparing the OP and the T2DM groups to the OA group.

**Conclusions:**

Our data indicates an altered bone remodelling rate in the T2DM group, which may, at least partially, explain the reduced bone strength and increased incidence of non-traumatic fractures in diabetic patients.

## Background

Type 2 diabetes mellitus (T2DM) and osteoporosis (OP) are two diseases with a high prevalence in developed countries. Both of these diseases are associated with a high morbidity and mortality rate and an increased risk of frailty fractures [1]. Hip fracture is the most devastating frailty fracture due to its associated high mortality and the low number of patients that recover functional activity and independence [2].

Various studies indicate an increased risk of fracture in T2DM. This risk varies depending on the type of study and the fracture location assessed. The incidence of frailty hip fractures in these patients is up to 2.8 times greater than in the general population [2]. Diabetes-related complications (retinopathy, neuropathy, kidney diseases, or vascular damages) may alter skeletal bone remodelling, since they impose a direct effect on bone strength and/or the probability of falls, thus increase the risk of fractures. Other aspects of diabetes, such as high levels of AGEs in bone collagen and/or hyperglycaemia in bone microenvironments, have also been considered as triggers to increased bone fragility [3]. Although many factors have been proposed, very few studies have analyzed the importance of each factor and the intimate mechanism of action on the deterioration of bone metabolism.

In bone formation, the signals that determine the differentiation, survival, and replication of osteoblastic cells are critical for suitable bone metabolism. Those signals that may be essential include: those involved in osteoblastic proliferation and differentiation, such as RUNX2 and OSTERIX (OSX); the signals involved in the osteoblast-osteoclast coupling process for bone remodelling, such as osteoprotegerin (OPG) and the receptor activator of nuclear factor-kβ ligand (RANKL); and the signals related to other common diabetic disease complications, such as AGE receptor (AGER). These signals may be affected in diseases, such as T2DM, due, at least in part, to common metabolic conditions in diabetic patients, such as high levels of glucose and/or AGEs. In this regard, there have been previous studies in animal models, in cell lines, and in primary human osteoblast (hOB) cultures [4−10] but, to the best of our knowledge, no previous studies have been carried out in hOB cultures from cancellous bone biopsies of diabetic patients.

The aim of this study is to assess the influence of T2DM on bone metabolism. For this purpose, primary human osteoblast-like cells were stimulated by using solely a high glucose concentration, and by using a high glucose concentration plus advanced glycation end products (AGEs), which are similar to the conditions found in diabetic patients.

## Methods

### Study design and subjects

The Ethics Committee of the *Virgen Macarena* Hospital approved this cross-sectional study, and informed written consent was obtained from all participants. All patients included in the study agreed to donate their bone samples.

The studied population consisted of three groups of patients. The first group consisted of 12 patients (1 man/11 women; 85 ± 9 years old) with osteoporotic fractures and T2DM (T2DM). The second group consisted of 10 patients (5 men/5 women; 81 ± 6 years old) with osteoporotic fractures (OP) without DM. The last group consisted of 10 patients (3 men/7 women; 68 ± 11 years old) with osteoarthritis (OA) without DM. The osteoarthritic samples (OA) were considered the reference group because the bone mineral density (BMD) values classified them as non-osteoporotic. The inclusion criterion for all of the groups was that patients must be aged over 50. The inclusion criteria for T2DM patients were the following: a) diagnosed with DM for more than 5 years; and b) a current frailty hip fracture (a fall from less than the patient’s height without an acceleration mechanism). For the OP group, patients had to have a frailty hip fracture without diabetic syndrome. Finally, OA patients could not have been previously diagnosed with osteoporosis or diabetes nor could they have a history of frailty fracture since the age of 50. The exclusion criteria for all of the groups included malignant diseases, hyperthyroidism, hyperparathyroidism, osteomalacia, previous bisphosphonate or systemic steroid treatment, and current use of hormone replacement therapy or any other drugs with negative effects on bone metabolism. All T2DM patients were being treated for diabetes, primarily with metformin, and only two patients were being treated with insulin.

Patients were included in the study in a consecutive manner (February 2013 to February 2014). Arthroplasty was performed in the Orthopaedics & Traumatology Department of the *Virgen Macarena* University Hospital (Seville, Spain) for all groups.

Standardized interviews and self-reported questionnaires were used to obtain the following information: age (years), weight (kg), height (cm), body mass index (BMI) (kg/m^2^), use of calcium and vitamin D supplements (none vs. any), use of medication, and family history of hip fracture.

### Bone mineral density (BMD)

Total hip and femoral neck BMD was measured at the contralateral hip using a dual X-ray densitometer (Hologic-Discovery, Hologic Inc.). The in vivo CV was 1 % (total BMD).

### Biochemical serum measurements

Fasting morning blood was drawn and stored at −80 °C. We assessed glucose, glycated haemoglobin (HbA1c), creatinine, cholesterol, triglycerides, calcium, phosphorus, and total alkaline phosphatase (AP) using an autoanalyzer (ADVIA 2400, Siemens; inter-assay CVs were 1.4, 1, 3.7, 1.2, 2, 3.7, 1.5, 1.5, and 3 %, respectively). Insulin-like growth factor I (IGF-I) was determined using a chemiluminescence immunoassay (CLIA) by autoanalyzer (IMMULITE 2000, Siemens; inter-assay CV was 6.9 %). Vitamin D [25(OH)D3] and the parathyroid hormone (PTH) were determined by CLIA using an immunoassay analyser (CP ADVIA Centaur, Siemens; inter-assay CVs were 20 and 4.3 %, respectively). Carboxy-terminal telopeptide of type I collagen (β-CTX) and procollagen I N-terminal peptide (P1NP) were analyzed by an electro-chemiluminescence immunoassay (ECLIA) using an autoanalyzer (ADVIA 2400, Siemens; inter-assay CVs were 2.9, 3.3 %, respectively). In all cases, the intra-assay CV was < 5 %.

### Cell culture and treatment with glucose and AGEs

During the total hip replacement, the femoral head was removed. Cancellous bone was cut into small pieces (1–2 mm) and placed in culture dishes. Bone pieces were gently washed several times with phosphate-buffered saline (PBS; Lonza) and nourished with Dulbecco’s modified eagle medium (DMEM) of 4.5 mM glucose containing 20 % heat-inactivated foetal bovine serum (FBS; Lonza) and 1 % Penicillin/Streptomycin/Fungizone (PSF) (Penicillin 100 U/mL and Streptomycin 100 mg/L (Sigma); Fungizone 250 μg/mL), and kept at 37 °C in a humidified atmosphere of 95 % air and 5 % CO_2_. After 7 days, the cells began to migrate from bone pieces and the culture medium was replaced every 2 to 3 days, at 80 % confluence of cells. The cells were then passaged with a trypsin-EDTA solution (Sigma) and plated in 6-well dishes at a density of 0.3×10^6^ cells/well for subsequent experiments. At 80 % sub-confluence, hOBs were growth-arrested for 24 h in serum-free DMEM containing 1 % PSF, and were then stimulated. To determine the effects of high glucose, either alone or together with AGE conditions on osteoblastic cells, cultures were cultivated in DMEM containing physiological glucose level (4.5 mM) or containing high levels of glucose concentration (25.5 mM), or high glucose plus AGEs (25 mM glucose and AGEs of 2 μg/ml) for 24 h. Osteoblast-like cell cultures were obtained from OA patients (*n* = 10), OP patients (*n* = 10), and T2DM patients (*n* = 12). The number of cells (index of proliferation) was counted with a haemocytometer. The trypan blue stain was used to screen dead cells after harvesting. Characterization of the osteoblastic phenotype was performed by measuring the alkaline phosphatase activity as previously described [11], with staining in all cultures. More than 85 % of the cells were positive for alkaline phosphatase.

Physiological glucose dosage (4.5 mM) was used as a control condition. High glucose and AGE dosages and time of exposure were chosen based on previous studies [5, 9, 12]. These concentrations are similar to those found in decompensated diabetic patients.

### Real-time PCR

Total RNA was isolated using the High Pure RNA Isolation Kit (Roche), according to the manufacturer's instructions. RNA concentration and purity were determined by A260 and A280 (A260/A280 = 1.7–2.0) measurements using a GeneQuant spectrophotometer (Amersham Biosciences. Cambridge, U.K.). Reverse transcription of total RNA (1 μg) was performed with the QuantiTec Reverse Transcription Kit (Qiagen) in accordance with the manufacturer’s instructions. The cDNAs were used for qRT-PCR analysis and all probes were analyzed for genes of interest (in duplicate): RUNX2, OSX, OPG, RANKL, and AGER. The QuantiTect Primer Assays were purchased from Qiagen: QT00020517 (RUNX2), QT00213514 (OSX), QT00014294 (OPG), QT00215614 (RANKL), QT00996625 (AGER), and QT00199367 (18S ribosomal). The cDNAs were amplified with SYBR Green PCR Master Mix (Sigma-Aldrich) in a StepOnePlus™ Real-Time PCR System (Applied Biosystems). The data was normalized to 18S ribosomal gene expression to obtain ΔCt. The 2^−ΔΔCt^ method was employed to calculate the gene expression differences between groups and the stimulated vs. physiological glucose. The results are expressed as arbitrary unit ± SEM.

### Statistical analysis

The results were analyzed using the SPSS 22.0 statistical software. Comparison of anthropometric data was performed using the two-way ANOVA test. Gene expression analysis was performed using the univariate general linear model (full factorial model) for inter-group comparisons, and an analysis of a general linear model with repeated measures (full factorial model) was used for intra-group comparisons. An adjustment for age was applied when needed. Correlation between variables was carried out by Pearson and Spearman’s Rho coefficient. The results with a *p* < 0.05 were considered statistically significant.

## Results

T2DM patients were older (85 ± 9 years) than OP (81 ± 6 years) and OA (68 ± 11 years) patients (Table 1). Due to these differences, an adjustment for age was applied to the successive statistical analyses. No differences were found in weight, height, or BMI between the study groups (Table 1).

**Table 1:** Anthropometric and BMD characteristics of the population studied (values are expressed as the mean ± standard deviation)

	OA (*n* = 10)	OP (*n* = 10)	T2DM (*n* = 12)	*p* value
Age (years)	68 ± 11	81 ± 6	85 ± 9	*0.009 **0.001
BMI (Kg/m^2^)	33.27 ± 5,15	27,91 ± 5,93	29,73 ± 5,74	–
Femoral neck BMD (gHA/cm2)	0.72 ± 0.19	0.52 ± 0.11	0.47 ± 0.03	–
Total hip BMD (gHA/cm2)	0.89 ± 0.14	0.77 ± 0.169	0.62 ± 0.103	–
Femoral neck T-score	−1.39 ± 1.41	−3.10 ± 0.87	−3.40 ± 0.26	–
Total hip T-score	−0.76 ± 0.79	−1.68 ± 1.07	−2.63 ± 0.83	–

*OA vs. OP; **OA vs. T2DM

Total hip and femoral neck BMD in both fractured groups, OP and T2DM, were fewer than the OA group. The T2DM group presented the lowest BMD values; however, after adjusting for age, these results were not significantly different (Table 1).

The biochemical profile of the three groups of patients (Table 2) was very similar, and we only observed differences in HbA1c (T2DM: 6.12 ± 0.77 % vs. OP: 5.20 ± 0.28 %, *p* = 0.011; T2DM vs. OA: 5.18 ± 0.29 %, *p* = 0.002). We also found major differences in the vitamin D levels. All of the groups showed mean vitamin D values lower than 20 ng/ml, but the OP group had the lowest values (OP: 7.62 ± 3.09 ng/ml vs. OA: 17.58 ± 11.45 ng/ml, *p* = 0.01). The bone turnover markers assessed were P1NP, β-CTX, and AP. We found greater mean β-CTX values in the two fracture groups compared with the control group, especially in the OP group (OP: 0.72 ± 0.22 ng/ml vs. OA: 0.36 ± 0.20 ng/ml, *p* = 0.022). There were no differences in P1NP serum levels among the groups, nor were there differences in AP levels.

**Table 2:** Biochemical parameters of the population studied (values expressed as the mean ± standard deviation)

	OA (*n* = 10)	OP (*n* = 10)	T2DM (*n* = 12)	*p* value
HbA1c (%)	5.18 ± 0.29	5.20 ± 0.28	6.12 ± 0.77	**0.002 ***0.011
Glucose (mg/dl)	88.30 ± 29.4	109.17 ± 34.1	117.40 ± 37.8	–
Cholesterol (mg/dl)	168.33 ± 44.0	115.33 ± 28.3	148 ± 25.4	*0.029
Triglycerides (mg/dl)	110.44 ± 44.0	107.33 ± 31.5	130.3 ± 37.2	–
Creatinine (mg/dl)	1.06 ± 0.56	1.17 ± 0.41	0.86 ± 0.17	–
Calcium (mg/dl)	9.47 ± 0.51	9.27 ± 0.43	9.37 ± 0.44	–
Phosphorus (mg/dl)	3.18 ± 0.53	2.98 ± 1.01	2.94 ± 0.54	–
IGF-1 (ng/ml)	67 ± 35.15	45.33 ± 26.57	43.5 ± 18.35	–
Vitamin D (ng/ml)	17.58 ± 11.45	7.62 ± 3.09	12.38 ± 7.72	*0.01
PTH (pg/ml)	42.94 ± 20.61	70.33 ± 43.64	64.90 ± 49.74	–
P1NP (ng/ml)	48.38 ± 25.10	54.49 ± 16.23	54.78 ± 38.61	–
β-CTX (ng/ml)	0.36 ± 0.20	0.72 ± 0.22	0.54 ± 0.30	*0.022
AP (UI/l)	172.2 ± 73.79	211.17 ± 36.47	192.9 ± 80.50	–

*OA vs. OP; **OA vs. T2DM; ***OP vs. T2DM

The proliferation of hOB in cultures from the T2DM patients was the slowest, and all of the cultures demonstrated different times for their confluence (32 ± 10 days for T2DM; 30 ± 11 days for OP; 21 ± 4 days for OA).

### Gene expression

**Fig. 1:**
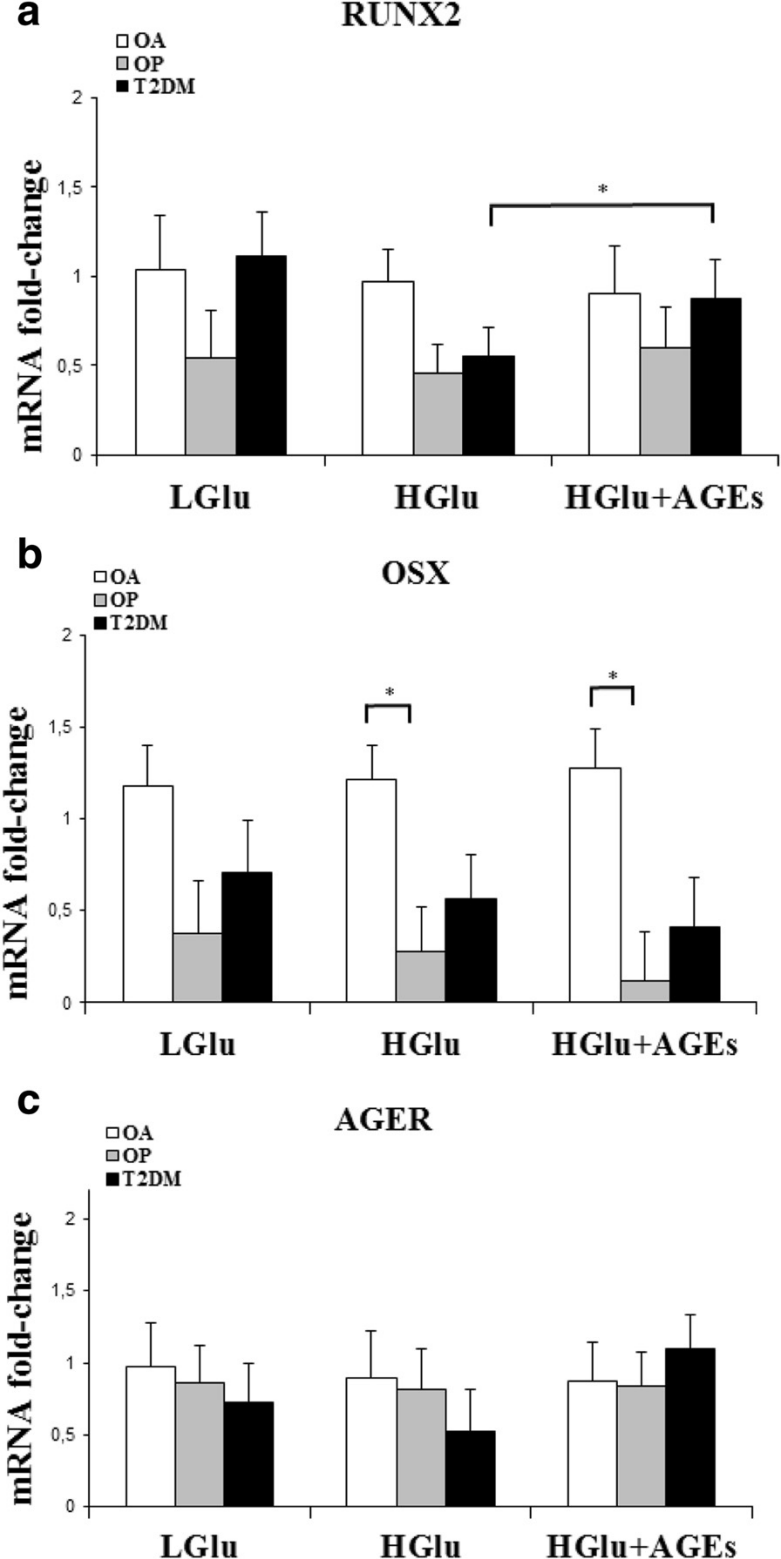
Gene expression of RUNX2 **a** OSX **b** and AGER **c** Osteoblasts from OA patients (*n* = 10), OP patients (*n* = 10) and T2DM patients (*n* = 12) were treated with low glucose as control (4.5 mM; Glu), high glucose (25 mM; HGlu) and high glucose + AGEs (2 μg/ml; HGlu + AGEs) during 24 h. The results were normalized to 18S ribosomal and adjusted for age in the statistical analyses. The data is expressed as mean ± SEM (relative units). (*statistically significant at *p < 0.05*)

**Fig. 2:**
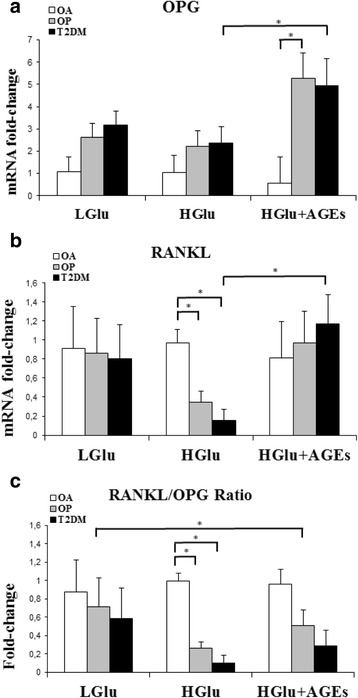
Gene expression of OPG **a** and RANKL **b** and the RANKL/OPG ratio **c** Osteoblasts from OA patients (*n* = 10), OP patients (*n* = 10) and T2DM patients (*n* = 12) were treated with low glucose as control (4.5 mM; Glu), high glucose (25 mM; HGlu) and high glucose + AGEs (2 μg/ml; HGlu + AGEs). The results were normalized to 18S ribosomal and adjusted for age in the statistical analyses. The data is expressed as mean ± SEM (relative units). (*statistically significant at *p < 0.05*)

The expression of genes encoding RUNX2, OSX, AGER, OPG, and RANKL were compared between those of the fractured groups (OP and T2DM) and of the OA group and referred to physiological glucose (Figs. 1 and 2). The results were reported in relative units of expression (r.u.). An adjustment for age was implemented in the statistical analyses.

RUNX2 expression levels (Fig. 1a) were constant in all of the culture conditions for the OA and OP groups, although the OP group consistently demonstrated lower levels (by approximately 50 %) than the OA group. In the T2DM group, the RUNX2 expression was similar to that of OA, but was lower (by approximately 50 %) in conditions of high glucose concentration. In the diabetic group, the expression of RUNX2 in conditions of high glucose was significantly lower than this expression in high glucose plus AGEs (HGlu: 0.550 ± 0.160 r.u., HGlu + AGEs: 0.872 ± 0.224 r.u.; *p* = 0.042).

The OSX gene expression (Fig. 1b) was markedly lower in both groups of patients with hip fracture than in the control group, and the OP group had the lowest values for all conditions. High glucose and high glucose plus AGEs reduced the OSX gene expressions in the two groups of patients with fractures: OP and T2DM. The mRNA levels were significantly lower in the case of the OP group compared with those of the OA group for the two treatments (OP vs. OA. HGlu, *p* = 0.033; HGlu + AGEs, *p* = 0.015).

At low glucose, AGER expression values (Fig. 1c) were slightly lower in the two pathologic groups than in the control group. This difference was accentuated when the cultures were subjected to a high glucose medium, with lower expression (28.11 %) in the diabetic group. In the case of the high glucose medium plus AGEs, the OP group showed no change from the baseline or from the high glucose condition. However, the T2DM group presented a higher AGER expression compared with the values observed in the low and high glucose experimental conditions (34 and 52.56 %, respectively).

The OPG gene expression (Fig. 2a) was greater in the OP and T2DM groups compared with those in the OA group for all the experimental conditions. We found no difference between the groups in a high glucose condition. In contrast, the high glucose medium plus AGEs increased the OPG expression levels in the two fractured groups with an increase of 35.71 and 50.27 % with respect to low glucose, and an increase of 57.62 and 52.02 % with respect to high glucose (OP and T2DM, respectively). The difference in expression when the AGEs were added to the high glucose medium was significantly greater in the OP group than in the OA group (OP vs. OA, *p* = 0.041) and was also significantly greater in the T2DM group when subjected to high glucose (HGlu. vs. HGlu + AGEs, *p* = 0.046).

The RANKL levels of expression (Fig. 2b) in low glucose were similar in all groups and remained stable under all culture conditions in the OA group. A significant reduction of the RANKL expression was observed in a high glucose medium for the two fractured groups compared with the OA group (OA vs. OP, *p* = 0.012; OA vs. T2DM, *p* = 0.002). In a high glucose medium plus AGEs, the two fractured groups showed similar expression values as those in the OA group. However, the diabetic group had a significantly greater expression (69.46 %) than the expression observed in high glucose conditions (HGlu vs. HGlu + AGEs, *p* = 0.015).

The RANKL/OPG ratio values (Fig. 2c) in the two fractured groups were lower than in the OA group for all culture conditions. In the case of high glucose, the ratio in the two fractured groups fell by 63.18 and 82.53 % (OP and T2DM, respectively) with respect to low glucose, with the two measures being significantly less than those in the OA group. Finally, the RANKL/OPG ratio of the OP group in a high glucose medium plus AGEs was significantly less than in low glucose (Glu vs. HGlu + AGEs, *p* = 0.02).

Mannitol, which was used as an osmolarity control, had no effect on the hOB cells (data not shown). Gene expression failed to correlate with age, BMI, or the biochemical parameters analyzed.

## Discussion

It is well accepted that diabetes mellitus (DM) increases the risk of fragility fractures. The mechanisms by which such impairments occur remain unclear, but long-term exposure to high glucose levels in patients with T2DM may lead to decreased bone remodelling which, in turn, may lead to structural deterioration. This could explain the well-documented higher risk for fracture despite preservation or an increase in BMD in patients with type 2 diabetes [1–3, 13, 14]. We have studied whether high glucose alone or, with the addition of AGEs in the primary culture of osteoblast-like cells (hOB) from diabetic patients, is able to promote changes in the proliferation, differentiation and/or function of these cells as a possible explanation for bone fragility in T2DM patients. For comparison purposes, we analyzed the effect of these treatments on cultures of hOB from patients with osteoarthritis as a control group [11].

The hOB cultures from the patients with hip fractures had slower proliferation rates than those from the OA group, and those from T2DM patients had the slowest proliferation rates. Due to the age difference between the groups, proliferation was not statistically different. These results are consistent with the idea that osteoblasts from OP patients are characterized by a slower growth [11] and that high glucose reduces osteoblast viability and proliferation [15–17].

The differentiation of osteoblasts is regulated by a network of interacting transcription factors, with Runx2 as the key player. In osteoblasts, this factor acts by binding to OSE2, in the promoter region of many genes crucial for osteoclast regulation, such as osteoprotegerin (OPG) and the receptor activator of nuclear factor-kβ ligand (RANKL). Runx2, and its target genes, also constitute crucial components of the organic matrix formation, such as osteocalcin, osteopontin and collagen type I alpha 1. Similarly, Osterix (OSX) is also an essential transcriptional factor for the differentiation of osteoblasts and matrix mineralization. Osx acts downstream of Runx2, and requires its signalling in order to be expressed. Osx drives osteoblasts to the late stage of maturation, and inhibits its proliferation by regulating the Wnt/β-catenin canonical pathway [18].

It is well known that Runx2 and Osx are key osteogenic transcription factors that are required for osteoblastic differentiation from mesenchymal stem cells (MSCs) [6]. Our results showed that hOB from T2DM patients were negatively affected with respect to RUNX2 and OSX gene expression when subjected to high glucose alone or together with AGEs in the culture medium, which is consistent with the data reported by other authors [4–8]. Recently, a down-regulation in RUNX2 and OSX expression has been demonstrated in pre-osteoblastic SCP-1 cells treated with serum from T2DM patients [19]. However, an increase in the expression of these genes [9, 18] or no variation in the expression [20] have also been reported by other authors. These apparently contradictory effects of high glucose in the various studies on the osteoblastic differentiation process may be due to the heterogeneity of the cell sources and to differences in the experimental procedures tested. A down-regulation or lack of RUNX2 and/or OSX is generally associated with a lower proliferation and differentiation of osteoblastic cells [21, 22].

OPG/RANK/RANKL is the most important intercommunication system between osteoblasts and osteoclasts, and many of the systemic drugs, cytokines, and growth factors influencing bone remodelling act on bone through this system. Acting as a decoy receptor, OPG blocks RANKL action and thereby balances bone turnover [23, 24]. The expression of OPG in basal glucose was greater in the hOB cultures from both fractured groups, OP and T2DM, whereas RANKL expression was similar in all 3 groups. However, high levels of glucose induced a major decrease in the expression of RANKL in the OP group and even more in the T2DM group, but OPG expression was not modified in any groups. These results indicate that high glucose suppresses bone resorption by decreasing RANKL expression, a decrease that seemed to be alleviated by the overstimulation of AGER when AGEs were added, as noticed by other authors [25]. In this regard, previous studies performed in osteoblastic and osteocyte cell lines [26] and in diabetic animals have demonstrated inconsistent results, showing both an increase and a decrease in the expression of OPG and RANKL [7, 9, 27] or no variation whatsoever [6]. The treatment with a high glucose medium plus AGEs produced a significant increase in OPG expression in the hOB cultures from the OP and T2DM groups. High OPG levels have been linked with macrovascular and microvascular damage in DM patients [28, 29]. Furthermore, a strong increase in OPG expression might cause a decrease in the bone remodelling cycle and contribute towards the vascular calcification that is so often found in diabetic patients [30].

When the RANKL/OPG ratio was analyzed, which is a potential osteoclastic marker, we observed that both treatments (HGlu and HGlu + AGEs) produced a notable reduction in both the OP and T2DM groups. Previous studies have pointed out that there is an imbalance in the RANKL/OPG ratio in the diabetic disease [6, 7, 9, 27]. To the best of our knowledge, no previous studies have been performed in hOB cultures from diabetic patients, although high OPG and low RANKL serum levels have been reported in patients with type 1 and type 2 diabetes [28, 29]. In this study, we simulated the metabolic conditions of poorly controlled T2DM patients, such as hyperglycaemia and high AGEs, in the hOB cultures.

Finding a decreased RANKL/OPG ratio implies a suppressed bone turnover process. Over the years, this deleterious effect on bone and the diminished ability for bone formation represent the origin of the greater frailty of diabetic bone.

In prolonged hyperglycaemia found in advanced and poorly controlled diabetes mellitus, glucose reacts with proteins to form advanced glycation end products (AGEs) [10]. The coupling of AGEs with the receptor (AGER) on osteoblastic lineage cells results in a reduction of the antioxidant mechanisms and increased synthesis of free-oxygen radicals, which coincides with the increased expression of RANKL and promotes the differentiation and activation of the osteoclasts and bone resorption [25] and induces the osteoblast apoptosis [31]. Additionally, elevated levels of AGEs alter the mineralization and repair of microdamage in bone tissue through the formation of crosslinks in collagen, which alters the bone mechanics [32, 33]. The natural accumulation of AGEs with age increases in diabetes and chronic inflammatory diseases, which points to AGEs as an essential factor in the impairment of bone quality in T2DM.

When we analyzed AGER expression levels, no significant changes were found. However, AGEs, in addition to high glucose, induced a slight increase in AGER expression, as observed by other authors [34]. This increased expression may contribute towards decreased bone formation since increased AGER produces an inhibition of osteoblast proliferation by depressing Wnt signalling via PI3K and ERK and by suppressing OSX expression [10, 35]. It is expected that poorly controlled diabetes patients have high circulating levels of AGEs for prolonged periods throughout their lifetime, which induces a more striking increase in AGER expression than found in our experiment. Our patients practised very good metabolic control of their diabetes and were primarily treated with metformin. This anti-diabetic drug has been reported to be able to reduce the AGER protein levels in osteoblast-like cell lines [17, 36]. Therefore, another reason for the low AGER mRNA values in the hOB cultures from diabetic patients could be due to a residual effect of bone adaptation to metformin in these patients, because eight of these patients were taking this drug for at least 1 year prior to hip fracture. Nevertheless, the role of the AGE receptor in bone fragility in diabetic patients has yet to be fully ascertained.

The study had several limitations. Regarding gene expression of osteoblast-like cell cultures, the results presented a large intra-group variability. This may be the reason why some of the observed intra- or inter-group differences showed certain tendencies but did not attain statistical significance. Another limitation was that we focussed on gene expression of the cells and the mineralization capacity was not assessed. It is possible that high glucose and/or AGE levels may also have an effect on the differentiation and function of osteoclasts, but this has not been assessed in this study. Future studies should examine the effects of high glucose and/or AGEs on these cells solely and/or co-cultured with osteoblasts. Another limitation of the study was the good metabolic control of diabetes found in our T2DM patients, as demonstrated by the HbA1c and triglyceride serum levels and by the fact that there were no additional complications. Despite these drawbacks, a strong point of this study is the use of primary cultures of human osteoblasts, which are an excellent model and are better than cell lines or animals for the evaluation of human bone pathologies.

## Conclusions

Our study provides the first experimental evidence of the deleterious effect of high levels of glucose and AGEs in RUNX2 and OSX expression. Moreover, T2DM osteoblasts demonstrate the greatest alteration in the OPG/RANKL system. Taken together, these alterations lead to an altered bone remodelling rate in the T2DM, which may, at least partially, explain the reduced bone strength and increased incidence of non-traumatic fractures in these patients.

## Abbreviations

AGER: Advanced glycation end products receptor
AGEs: Advanced glycation end products
AP: Alkaline phosphatase
BMD: Bone mass density
BMI: Body mass index
cDNA: Complementary deoxyribonucleic acid
DM: Diabetes mellitus
DMEM: Dulbecco’s modified eagle medium
HbA1c: Glycated haemoglobin
hOB: Human osteoblasts-like cells
IGF-I: Insulin-like growth factor I
OA: Osteoarthritis
OP: Osteoporosis
OPG: Osteoprotegerin
OSX: Osterix
P1NP: Procollagen I N-terminal peptide
PBS: Phosphate-buffered saline
PSF: Penicillin/streptomycin/fungizone mix
PTH: Parathyroid hormone
RANK: Receptor activator of nuclear factor kβ
RANKL: Receptor activator of nuclear factor kβ ligand
RNA: Ribonucleic acid
T2DM: Type 2 diabetes mellitus
β-CTX: Carboxy-terminal telopeptide of type I collagen
